# A cautionary note on the appropriateness of using a linkage resource for an association study

**DOI:** 10.1186/1471-2156-4-S1-S89

**Published:** 2003-12-31

**Authors:** Kristina Allen-Brady, James M Farnham, Jeff Weiler, Nicola J Camp

**Affiliations:** 1 Genetic Epidemiology, Department of Medical Informatics, University of Utah School of Medicine, Salt Lake City, Utah, USA; 2 Eccles Health Sciences Library, University of Utah, Salt Lake City, Utah, USA

## Abstract

**Background:**

Utilizing a linkage resource for association analysis requires consideration both of the marker data used and correlations among relatives in pedigrees. We previously developed a method for association testing in pedigrees. We applied our method to 50 replicates of microsatellite data surrounding five genes involved in high-density lipoprotein (HDL) in the Genetic Analysis Workshop 13 (GAW13) simulated data and examined association with HDL as well as linkage disequilibrium (LD) between markers.

**Results:**

Although no association was intentionally simulated, we found significant evidence of weak LD between microsatellite markers (flanking/~5 cM from the genes), in some but not all replicates. This level of LD compared well to that observed in the real GAW13 Framingham data. Only one region had sufficient replicates to assess power, and this was low (12.5–20.8%). More power was attained using all individuals and accounting for relationships, compared with one independent individual/pedigree, although this was not significant due to small sample sizes. Not accounting for relatedness inflated statistical significance (p < 0.0001).

**Conclusion:**

A correction for dependence is necessary in association studies to avoid an inflation of significance probabilities. Our results further illustrate that use of microsatellite marker data is not an effective approach for association testing.

## Background

Association and linkage disequilibrium analyses are tools that are often used to fine-map and refine promising linkage findings. These tools are particularly effective because they increase power to detect genes with small effects [1]. Many pedigree resources ascertained for linkage studies exist. However, the use of a linkage resource for an association analysis requires not only a methodology to address the issue of correlations among relatives in extended pedigrees but also consideration of the appropriateness of using microsatellite marker data previously genotyped for linkage analyses.

There is great potential for the utilization of pedigree resources for association analyses. Our group and others have developed methodologies to enable association analyses to be performed in extended pedigrees [2,3]. If multiple related individuals are included in a study without accounting for the relationships among them, there is an underestimate of the variance, which leads to an increased probability of a type I error. Slager and Schaid [2] approached the problem from a theoretical perspective and derived the correction necessary for a case-control association test. Using identity by descent (IBD) probabilities from a linkage analysis on marker data using GENEHUNTER [4], their method corrects the variance estimate to produce a correct significance assessment. Unfortunately, in moderate to large pedigrees, the calculation of the necessary IBD probabilities cannot be done, since GENEHUNTER is limited in the pedigree size it can analyze. Our method can analyze pedigrees of arbitrary size by using an empirical approach to provide a valid statistical test for association [3].

For each simulation, our program uses a gene-drop to generate null genotype configurations for a given set of individuals in pedigrees. The gene-drop requires the assignment of genotypes to the pedigree founders, based on allele frequencies estimated from the study population. This is followed by the use of Mendelian inheritance probabilities to determine the genotypes of all descendants. The resulting genotype configuration on each pedigree represents a possible configuration under the null hypothesis of no association between allele and disease. Using the simulated genotypes for individuals for whom real data are available and the true phenotype data, the statistic of interest is calculated (for example, in this study a chi-squared test for independence). The resulting statistic is from the null distribution because it was derived from data under the null hypothesis of no association. This procedure is repeated many times, creating an empirical null distribution. The real genotype data for the same individuals and real phenotype data are then used to calculate the observed statistic. This observed statistic is then compared with the empirical distribution to determine significance. The method can be applied to pedigrees of any structure and size and to any statistic of interest.

Association analyses are based on either testing the true disease-causing variant itself, or alleles at a marker in linkage disequilibrium with the true variant. Since linkage disequilibrium (LD) generally extends over very short distances (typically < 100 kb, [5]) and since markers with multiple alleles impose multiple testing, markers for association analyses are usually chosen to be intra-genic single-nucleotide polymorphisms (SNPs). In contrast, linkage markers are chosen to be highly polymorphic to maximize linkage informativeness and usually are at a resolution of 5–10 cM. Hence, marker choice for the two methods is distinct.

The Genetic Analysis Workshop 13 (GAW13) simulated data were modeled on the real GAW13 Framingham Heart Study data, as a genomic search with average resolution of approximately 9.5 cM, and hence no LD, or association, was intentionally simulated. To investigate the value, if any, of using linkage microsatellite marker data for association studies, we chose to analyze the simulated high-density lipoprotein (HDL) phenotype (dichotomized to the two extreme quartiles: high versus low quartile) and microsatellite markers either flanking or within approximately 5 cM of the true location of five genes involved in determination of baseline simulated HDL, as indicated in the 'answers'.

Here we report the results of our empirical simulation method using a chi-squared association analysis for genotype data in the GAW13 simulated data. Although no association was intentionally included in the GAW13 simulated data, we report the underlying linkage disequilibrium (LD) between microsatellite markers and the ability of our empirical association method to detect power and false-positive signals. As a comparison we also report the LD in the Framingham data for the same location as one of the simulated baseline HDL genes.

## Methods

The first 50 replicates of the simulated GAW13 data with missing genotype and phenotype values were utilized in this study. There were 330 families, ranging in size from 7 to 84 members, in each of the 50 replicates studied.

### Genotype data

Eleven baseline genes (b12, ...,b22) contribute to the HDL phenotype. We chose to study five of these genes (b13, b14, b16, b18, and b22), selected to represent a spectrum of percent contribution to the HDL trait (see Table 1). For each gene of interest, microsatellite markers either flanking (and <15 cM), or within ~5 cM of the true gene location were studied, resulting in two to four markers per gene region being analyzed. The average marker resolution ranged from 3.30 cM (b22) to 14.33 cM (b14). To reduce multiple testing, we selected only the three highest frequency alleles from each microsatellite marker for our analyses, and analyzed genotype distributions considering both dominant and recessive modes of inheritance.

### Phenotype data

For all analyses, we used the maximum HDL measurement for each individual across the longitudinal study period as the trait of interest. We selected the covariates sex, age (at maximum HDL), BMI (using mean height and weight across the study period), smoking (ever/never), alcohol (ever/never), and fasting glucose (ever/never > 126 mg/dl).

We considered the analysis of the HDL data in three different ways. First, we adjusted the maximum HDL value using the generating equation as provided in the 'answers' (*GAW13_HDL(PED)*). Second, we used our own linear regression equation to adjust HDL (*LR_HDL*) using the above listed covariates, where for each replicate we performed a linear regression with these covariates as independent variables to determine the regression coefficients. Third, we performed an 'independent' (*IND*) analysis by selecting only the first individual from each family with data and also using the GAW13 'answers' (*GAW13_HDL(IND)*).

### Linkage disequilibrium

The estimating haplotype (EH) program [6] was used to determine maximum-likelihood estimates of LD in all 50 replicates for markers in all five regions. Comparisons between all pair-wise combinations of the microsatellite markers in the regions of interest were performed. The difference between the maximum-likelihood values calculated by EH for the haplotype frequencies under H_1 _(allelic association allowed) and for the haplotype frequencies under H_0 _(no association) were used to calculate the raw disequilibrium, from which D', the proportion of maximum possible disequilibrium, was determined. Significance was determined by the EH program.

To compare results of the simulated data to the GAW13 Framingham data, D' for the three most common alleles at the first four markers on chromosome 4, corresponding to the exact position of the four markers surrounding gene b22 in the simulated data set, were analyzed.

It should be noted that the simulated data were generated to contain linkage to baseline HDL genes, but not LD or association with particular gene variants in the candidate genes. However, linkage and association differ only in the fact that the former is a phenomenon of loci and the latter of alleles at loci. In replicates where LD exists across markers, the simulated linkage creates a scenario equivalent to allelic heterogeneity for association. While certainly not ideal, allelic heterogeneity is a reasonable model for real data in which multiple common variants within a gene may increase disease susceptibility.

### Statistical analyses

In all analyses we compared the highest and lowest quartiles of the HDL phenotype of interest. We used the chi-squared statistic for independence considering both dominant and recessive models as our statistic of interest. For each analysis 10,000 simulations were generated to create the empirical null distribution. To illustrate the necessity to correct for relatedness, we compared the results from *GAW13_HDL(PED) *with an analysis where we ignored genealogy and included all individuals (*ALL*) with data in the pedigrees without any correction for relatedness. A paired t-test for the average of the -ln(p) across markers over the 50 replicates was used to compare the results.

We report the number of replicates showing LD at various levels of significance for each gene region. Only one region surrounding gene b22 had sufficient replicates with significant LD to assess power. For this region we also show the percentage of the replicates with significant LD for which a significant association was found (*p *< 0.01) for each of the three analysis types, indicating a power estimate for each. Two regions (surrounding b14 and b18) had sufficient replicates without LD (*p *> 0.5) to assess false-positive findings. For these regions we show the proportion of replicates without LD for which significant associations were found (*p *< 0.01).

All regression analyses (for *LR_HDL*) and classic chi-square analyses (for *GAW13_HDL(IND)*) were performed using STATA 6.0 (College Station, Texas). Fisher's exact *p*-values are reported for the *GAW13_HDL(IND) *analyses, where appropriate.

## Results

For the 'independent' (*IND*) analysis the average sample size per replicate with data was 328.7 individuals and ranged between 324 and 330 individuals. For all other analyses using all individuals with available data, the average sample size per replicate was 1672.6 individuals and ranged between 1627 and 1715. More than 95% of families within the 50 replicates had two or more members per family with data.

### Empirical method versus all individuals/pedigree without correction

A comparison of the empirical method using *GAW13_HDL(PED) *with the uncorrected *GAW13_HDL(ALL) *showed extreme statistical significance (t = -43.86, *p *< 0.0001), suggesting that not accounting for correlations among family members substantially underestimates the variance and inflates the significance, as expected.

### Linkage disequilibrium

Although no association between microsatellite markers and the underlying genes was intentionally simulated, for every gene studied significant LD (*p *< 0.05) was present in at least one replicate (Table 1). The region surrounding gene b22 had the most replicates with significant LD, with nearly half (24/50) of the replicates analyzed indicating LD.

For the GAW13 Framingham data on chromosome 4 (equivalent to those markers surrounding b22 in the simulated data), values of D' ranging from 9.7 to 47.6 were observed. These values compared well to those found for the simulated data (D' = 9.4–31.8).

### Power: association findings for the region containing b22

As the region containing b22 was the only one with significant LD present in sufficient replicates (24/50), we chose to further study power of the association analyses for only this region. Table 2 shows the number of replicates where a significant association (*p *< 0.01) was found using the various analyses. Power was low for all comparisons and ranged from 12.5–20.8%. Power was higher for analyses that corrected for covariates and that used pedigree data (average sample size = 1672.6, power = 20.8%), rather than 'independent' data (sample size = 328.7, power = 12.5%), although these differences are not statistically significant due to a small sample size (*n *= 24). However, 20.8 % is significantly different from 0.05 (type I error rate) as assessed by binomial distribution theory with *n *= 24 (*p *= 0.006).

### False positives: association findings for regions containing b14 and b18

The number of false-positive results was assessed in gene regions for which sufficient replicates were available and for which LD was not evident (*p *> 0.5). Two regions surrounding genes b14 and b18 had sufficient replicates (32/50 and 21/50, respectively) to assess false-positive findings. Results are shown in Table 3. By inspection, the number of replicates for which false positives were found differed at most by one across analyses. The observed rates of false-positive signals ranged from 0.048 (1/21) to 0.125 (4/32) and, due to small sample sizes, are not significantly different from each other, or from 0.05. These results are, of course, purely observational. To accurately assess false-positive rates thousands of replicates would be necessary.

## Conclusions

Association-based analyses using extended pedigree data require a method that accounts for correlations among relatives. The substantial proportion of multiple related individuals per pedigree in this study necessitates such a correction. In this study, we have shown, as expected, that not correcting for the relatedness of family members resulted in a sharp inflation of the significance probabilities. A valid test can be derived by sampling only one individual from each pedigree or by using a correction method, such as our empirical approach, which determines the empirical significance accounting for the relatedness of individuals. Our results are consistent with the hypothesis that the increased sample size in using multiple individuals from extended pedigrees and accounting for their relatedness can increase power.

Although no association was intentionally simulated in this data set, we found significant LD between microsatellite markers for several gene regions, particularly the region containing gene b22, which we chose to study power. The result that the b22 region contained the most LD is perhaps not unexpected because markers in that region were at the highest density (average 3.30 cM). In fact, the frequency of detectable LD in replicates correlated reasonably well with available marker density (see Table 1). However, the D' values were low, although approximately equal to those obtained in the real GAW13 Framingham data. Considering only the b22 region and replicates with significant LD, power was found to be low (<21%). Again, perhaps this is not unexpected because LD values were low and b22 contributed only 1% to the genetic variance of HDL.

Considering all five gene regions studied, 80% (4/5) of the regions did not exhibit a high frequency of LD over the 50 replicates. Furthermore, as pointed out above, even for the one region with significant LD, power was low. These results could be extrapolated to association analyses in the real GAW13 Framingham data where LD was comparable and where small effect genes are expected. This indicates caution in the interpretation of any positive association findings in those data.

Microsatellite markers are not an ideal marker type for association testing. We selected the nearest two to four markers for each gene (each either flanking or within ~5 cM of the gene of interest). However, 5 cM represents a large distance between markers and true gene variants for association testing. Testing single-nucleotide polymorphism (SNP) variants within the gene, which are more likely to be in strong LD with disease-causing variants, would have been a more powerful situation.

A limitation of our analysis is that not all alleles were analyzed. Only the three highest frequency alleles for each marker were used to both increase the chance of an allele being observed sufficiently for association testing and to reduce multiple testing problems. This modified allele system is not biased for investigating LD, since alleles were not grouped according to their respective disequilibria. Further, we used the same allele definition for LD estimation and association, thus they are directly relevant to one another. We may, however, have missed positive association findings by not testing all alleles. A further limitation is that these data were not simulated to specifically contain association, and our power will be limited by the situation equivalent to allelic heterogeneity, which has been shown to reduce power in association-based analyses [7]. In addition, our study assessed the extreme quartiles for simulated HDL, and not the quantitative measure that was available. Our empirical approach, although theoretically completely general for any statistic, is not currently developed to analyze quantitative measures. This dichotomization may have reduced power to detect association.

In conclusion, our results indicate that the empirical approach makes efficient use of data collected from extended pedigrees. Further, this study illustrates the lack of power available by using linkage microsatellite markers, and strongly cautions against doing so for association testing.

## Figures and Tables

**Table 1:** Linkage disequilibrium across 50 replicates.

	**Genes**				
	
	**b13**	**b14**	**b16**	**b22**	**b18**
Contribution to HDL trait	0.1	0.05	0.02	0.01	0.005
Average marker resolution	5.52	14.33	7.14	3.30	5.45
No. reps with LD (*p *< 0.05)	8	2	8	24	5
No. reps with LD (0.05 <*p *< 0.1)	6	2	6	13	2
No. reps with LD (0.1 <*p *< 0.2)	19	4	7	5	5
No. reps 'without' LD (*p *> 0.5)	3	32	6	0	21

**Table 2:** Power: association findings for the region containing gene b22.

**Method of Analysis**	**No. Replicates Indicating Association**	**% Replicates with LD at *p *< 0.05**
GAW13_HDL (PED)	5/24	20.8
GAW13_HDL (IND)	3/24	12.5
LR_HDL (PED)	5/24	20.8

**Table 3:** False Positives: association findings for regions containing genes b14 and b18.

**Method of Analysis**	**No. Replicates Indicating Association**	**% Replicates with LD at *p *< 0.05 (proportion)**
b14		
GAW13_HDL (PED)	4/32	0.125
GAW13_HDL (IND)	3/32	0.094
LR_HDL (PED)	4/32	0.125
b18		
GAW13_HDL (PED)	1/21	0.048
GAW13_HDL (IND)	1/21	0.048
LR_HDL (PED)	2/21	0.095
